# Mediterranean circulation perturbations over the last five centuries: Relevance to past Eastern Mediterranean Transient-type events

**DOI:** 10.1038/srep29623

**Published:** 2016-07-14

**Authors:** Alessandro Incarbona, Belen Martrat, P. Graham Mortyn, Mario Sprovieri, Patrizia Ziveri, Alexandra Gogou, Gabriel Jordà, Elena Xoplaki, Juerg Luterbacher, Leonardo Langone, Gianluca Marino, Laura Rodríguez-Sanz, Maria Triantaphyllou, Enrico Di Stefano, Joan O. Grimalt, Giorgio Tranchida, Rodolfo Sprovieri, Salvatore Mazzola

**Affiliations:** 1 Università di Palermo, Dipartimento di Scienze della Terra e del Mare, Via Archirafi 22, 90123 Palermo, Italy; 2 Department of Environmental Chemistry, Institute of Environmental Assessment and Water Research (IDÆA), Spanish Council for Scientific Research (CSIC), Jordi Girona 18, 08034 Barcelona, Spain; 3 University of Cambridge, Department of Earth Sciences, Downing Site, Downing Street, Cambridge CB2 3EQ, United Kingdom; 4 Universitat Autònoma de Barcelona (UAB), Institute of Environmental Science and Technology (ICTA), Edifici Z, Carrer de les Columnes, Campus de la UAB, 08193 Bellaterra (Cerdanyola del Vallès), Barcelona, Spain; 5 UAB, Department of Geography, 08193 Bellaterra (Cerdanyola del Vallès), Barcelona, Spain; 6 Consiglio Nazionale delle Ricerche (CNR), Istituto per l’Ambiente Marino Costiero, Via del Mare 3, 91021 Torretta-Granitola (Trapani), Italy; 7 Vrije Universiteit Amsterdam, Department of Earth Sciences, Faculty of Earth and Life Sciences, de Boelelaan 1085, 1081HV Amsterdam, The Netherlands; 8 ICREA, Catalan Institution for Research and Advanced Studies, 08010, Barcelona, Spain; 9 Hellenic Centre for Marine Research (HCMR), Institute of Oceanography, P.O. Box 712, 19013 Anavyssos, Greece; 10 Department of Ecology and Marine Resources, IMEDEA (CSIC-UIB), Institut Mediterrani d’Estudis Avançats, Miquel Marquès 21, 07190 Esporles, Illes Balears, Spain; 11 Justus-Liebig-University Giessen, Department of Geography, Climatology, Climate Dynamics and Climate Change, Senckenbergstr. 1, 35390 Giessen, Germany; 12 Centre for International Development and Environmental Research, Justus-Liebig-University Giessen, 35390 Giessen, Germany; 13 CNR, Istituto di Scienze Marine, Via Gobetti 101, 40129 Bologna, Italy; 14 Research School of Earth Sciences, The Australian National University, Canberra, Australian Capital Territory 2601, Australia; 15 University of Athens, Faculty of Geology and Geoenvironment, Department of Historical Geology – Paleontology, Panepistimiopolis 15784, Athens, Greece

## Abstract

The Eastern Mediterranean Transient (EMT) occurred in the Aegean Sea from 1988 to 1995 and is the most significant intermediate-to-deep Mediterranean overturning perturbation reported by instrumental records. The EMT was likely caused by accumulation of high salinity waters in the Levantine and enhanced heat loss in the Aegean Sea, coupled with surface water freshening in the Sicily Channel. It is still unknown whether similar transients occurred in the past and, if so, what their forcing processes were. In this study, sediments from the Sicily Channel document surface water freshening (SCFR) at 1910 ± 12, 1812 ± 18, 1725 ± 25 and 1580 ± 30 CE. A regional ocean hindcast links SCFR to enhanced deep-water production and in turn to strengthened Mediterranean thermohaline circulation. Independent evidence collected in the Aegean Sea supports this reconstruction, showing that enhanced bottom water ventilation in the Eastern Mediterranean was associated with each SCFR event. Comparison between the records and multi-decadal atmospheric circulation patterns and climatic external forcings indicates that Mediterranean circulation destabilisation occurs during positive North Atlantic Oscillation (NAO) and negative Atlantic Multidecadal Oscillation (AMO) phases, reduced solar activity and strong tropical volcanic eruptions. They may have recurrently produced favourable deep-water formation conditions, both increasing salinity and reducing temperature on multi-decadal time scales.

The Mediterranean Sea operates as a “miniature ocean” in which to test processes directly linked to deep-water formation and where changes can be amplified and accelerated with respect to the global ocean^1^. Until recently, the best-known Mediterranean deep-water formation regions were the Gulf of Lions and the Adriatic areas (Fig. 1A). However, an additional overturning cell has since been detected in the Aegean Sea during an important hydrographic shift, which has been termed the Eastern Mediterranean Transient (EMT)^2^. The EMT lasted from the late-1980s to the mid-1990s and appears as the major climatic perturbation of circulation and water mass properties of the Mediterranean since 1950s, when instrumental observations have been systematically available (refs 2, 3, 4, 5; Fig. 1B). The consequences of the process continue today in the Western Mediterranean, in the Tyrrhenian and Ligurian sub-basins^6^ with major impacts on the seawater physico-chemistry, including the vertical and spatial distribution of anthropogenic carbon^7^. Physical conditions linked to the onset of the EMT are, among others, high salinity seawater in the Aegean and Levantine sub-basins, strong upper stratification in the Cretan Channel, together with anticyclonic circulation in the Ionian-Adriatic exchange^2^,^3^,^4^,^8^,^9^,^10^.

Convection and air-sea heat exchanges in the Mediterranean are a source of energy and essential regulators of climate, and may ultimately be transferred to key sites of North Atlantic overturning circulation^1^,^11^,^12^,^13^,^14^. There is a wide interest in understanding whether Mediterranean circulation perturbations like the EMT are frequent or cyclical and how they may have altered Mediterranean/Atlantic interactions. However, the record of historic hydrographic observations is short and characterized by scattered and limited precision data before the 1950s^10^,^15^. Direct oceanographic-paleoceanographic comparison remains indeed an open scientific challenge. Oceanographic studies, although limited to the last few decades, benefit from very-high accuracy techniques and daily/monthly/annual measurements of a variety of parameters relevant to deep-water formation. Paleoceanographic reconstructions from sediment-based archives differ in terms of data sensitivity and affordable time-resolution, though their time averaging tendency allows simple hypothesis testing and model simulation comparison in the absence of direct instrumental data.

In this study, organic and inorganic geochemistry of marine sediments recovered in the Sicily Channel (St 342 and St 407; Fig. 1A) trace a multi-decadal return period of surface freshening over the last five centuries. These events are in line with enhanced deep ventilation in the Aegean, as recorded by organic compound analyses (Athos-M2; Fig. 1A; ref. 16). Climate model simulations and observations tested here suggest a link between Sicily Channel surface freshening episodes and periods of intensified Mediterranean deep water formation, including the well-known EMT during which a pronounced deep-water formation took place in the Aegean Sea. These episodes seem to be recurrent perturbations in the Mediterranean thermohaline circulation strength and parallel modifications in atmospheric circulation patterns, during periods of frequent volcanic eruptions and low solar irradiance.

## Results

### Geochemical data

**Figure 2:**
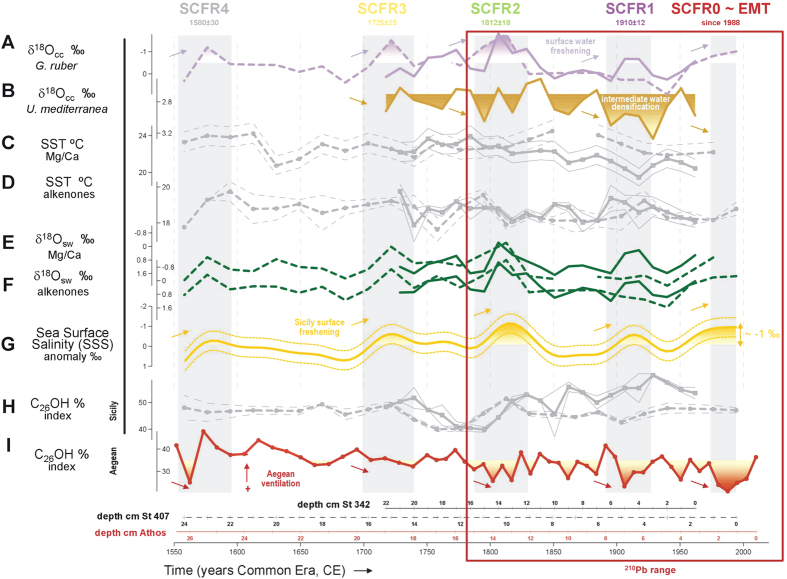
Paleo-dataset in Sicily and Aegean Sea sediments over the last five centuries. Time progression in years of the Common Era (CE). (**A**) δ^18^O_calcite_ of *Globigerinoides ruber* and (**B**) *Uvigerina mediterranea* reflecting Sicily Channel surface and bottom water properties, respectively. Sicily Channel SSTs derived from (**C**) the Mg/Ca ratio and (**D**) alkenone palæothermometers Normalized δ^18^O_sw_ profiles in the Sicily Channel after temperature correction of (**E**) *G. ruber* Mg/Ca-δ^18^O_calcite_ and (**F**) alkenones. (**G**) Resultant salinity anomaly, shown as a 20-year Gaussian low pass filter of the normalized δ^18^O_sw_ composite, based on reconstructed oxygen isotopic composition of seawater δ^18^O_sw_ in the Sicily Channel (details in Materials and Methods) derived from (**E**) and (**F**). Values above average (−0.1‰) are filled in. Relative proportion of n-hexacosan-1-ol (C_26_OH) to the sum of C_26_OH plus n-nonacosane (C_29_) in (**H**) the Sicily Channel and (**I**) the Aegean Sea sediments. The Eastern Mediterranean Transient (EMT) detected since1988 and similar past anomalies (SCFR1 1910 ± 12, SCFR2 1812 ± 18, SCFR3 1725 ± 25 and SCFR4 1580 ± 30) are shown by bands shaded in grey. In panels (**A–F**,**G**), dashed lines refer to St 407 and solid lines to St 342. Bands in panels (**C**,**D**,**H**) are drawn by using measurement replicates. Core depth-age equivalences are at the bottom of the figure. The box in red shows the time interval within the ^210^Pb range (see Fig. 4).

Different indicators were considered, as summarised in Fig. 2 (details of procedures in **Materials and Methods** and chronology of sediment cores St 342, St 407 and Athos-M2 in SI Appendix, Table S1 and Fig. S1). Firstly, oxygen isotope values of planktonic (*Globigerinoides ruber*) and benthic (*Uvigerina mediterranea*) calcite shells of foraminifera (δ^18^O_calcite_, in ‰) were measured in St 342 and St 407 to trace the pattern of hydrographic change. Shifts to lighter δ^18^O values recorded in the surface dwelling planktonic foraminifer can be seen in the Sicily Channel cores during the most recent EMT (since 1988) and in 1910 ± 12, 1812 ± 18, 1725 ± 25 and 1580 ± 30 (inverted axis, lighter upwards, Fig. 2A). A surface-to-deep contrast is traced in St 342, where benthic enriched values are observed for each past lightening event (Fig. 2B).

Secondly, past sea surface temperatures (SST, in °C) in St 342 and St 407 were estimated using two independent methods, *G. ruber* Mg/Ca values and di- and tri-unsaturated alkenones of 37 carbons. Mean values are warmer for the Mg/Ca reconstruction (22 ± 1 °C; Fig. 2C) than for the alkenone paleothermometer (19 ± 1 °C; Fig. 2D), given that *G. ruber* Mg/Ca reflects temperatures during summer-to-autumn^17^, and that alkenones are well suited to reconstruct the mean annual SST^18^. The SST estimated in the surface sediments of the Sicily Channel with these methods show a good match with present average SST data, which is 16 °C in winter-spring, and 23 °C and 22 °C during summer-autumn, respectively (annual mean ~19 °C).

The hydrographic nature of the past δ^18^O *G. ruber* variations is shown as an St 342/St 407 composite of the δ^18^O of seawater (δ^18^O_sw_) (Fig. 2E–G). The temperature effect is removed using the *G. ruber* Mg/Ca- and alkenone-derived SSTs (Fig. 2C,D) to extract the salinity component mainly. Each δ^18^O_sw_ time series is normalized (by subtracting its mean and dividing through its standard deviation) and gaps are filled in the normalized δ^18^O_sw_ profiles derived by *G. ruber* Mg/Ca-δ^18^O_calcite_ pairs (Fig. 2E) by using normalized alkenone-derived δ^18^O_sw_ data (Fig. 2F). Results are low-pass filtered using a Gaussian window and the “filtfilt” MATLAB algorithm, including estimation of the uncertainty associated with the δ^18^O_sw_ composite (further details in **Materials and Methods**). The δ^18^O_sw_ record documents a multi-decadal return period of surface freshening in the Sicily Channel over the last five centuries (inverted axis, less salinity upwards, Fig. 2G). We name these past perturbations as Sicily Channel freshening episodes (henceforth SCFR) and progressively number them from SCFR0 (coincident with the EMT) to SCFR4 (grey vertical bars in Fig. 2).

Given today’s Mediterranean-wide salinity range and precipitation variability, and the uncertainties in the conversion of paleo-δ^18^O_sw_ profiles to salinity reconstructions^19^, only qualitative estimates are provided here, that is, characterisation of the SCFR episodes as more- or less-pronounced than the EMT. The most recent EMT featured a 0.5 psu salinity change in the Sicily Channel^20^, which is revealed in the St 342/St 407 composite by a ~−1‰ δ^18^O_sw_ anomaly. A δ^18^O_sw_ decrease of similar amplitude can be ascribed to previous events, except for the oldest episode 1580 ± 30 (SCFR4), which is incomplete and subdued and the decade around 1812 ± 30 (SCFR2), which is slightly more intense than the EMT (Fig. 2G). The amplitude (larger than 1‰) of the main δ^18^O_sw_ variations in the Strait of Sicily, exceeding the propagated uncertainties of the various methods at the 95% confidence level, lends confidence that the observed δ^18^O_sw_ decrease stems from salinity reductions. Recent instrumental measures in the Mediterranean basin show that oxygen isotope ratios in meteoric precipitation (δ^18^O_precipitation_) vary closely in line with the surface salinity changes (88% of the variance explained, N = 204; Fig. S2). Following this relation, the EMT 0.5 psu salinity change reported in Gasparini *et al.*^20^ (from 37.7 to 37.2‰; red symbols in SI Appendix, Fig. S2), and preserved in foraminiferal isotopic ratios as a −1‰ δ^18^O_sw_ anomaly, would be approximately equivalent to a 0.1‰ decrease in δ^18^O_precipitation_ (from 1.3 to 1.2‰; Fig. S2). Finally, characterisation of the deep-water ventilation is provided by the relative proportion of *n*-hexacosan-*1*-ol (C_26_OH) to the sum of C_26_OH plus n-nonacosane (C_29_), an organic chemical index reflecting oxygenation variability^21^. This ratio was quantified in both the Sicily Channel (Fig. 2H) and the Aegean Sea sediments (Fig. 2I). During the EMT, the index suggests unchanged to reduced ventilation in the Sicily Channel while ventilation in the Aegean area increases, which would be consistent with instrumental observations of the mixed layer depth over the last decades^2^.

### Ocean modelling

**Figure 3:**
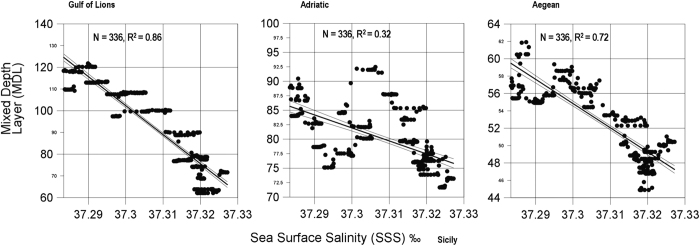
Scatter plots of surface salinity in the Sicily Channel and mixed layer depth in deep water formation sites (Gulf of Lions, Adriatic and Aegean) during winter. Data are obtained from the NEMOMED8 hindcast.

Mediterranean climate variability was also characterized using a regional ocean hindcast (NEMOMED8; 22,23). This hindcast has proven to be in good agreement with hydrographic gridded datasets based on observations and to correctly reproduce the sequence of events that lead to EMT and posterior evolution^22^,^23^. The mixed layer depth (MLD) from the hindcast is used as an indicator of deep-water formation strength. MLD shows a clear inter-annual variability with maximum values in February and robust correlation with the amount of deep-water formation in the Gulf of Lions, Adriatic and Aegean Seas. One of the main results from the model is the statistically significant correlation between surface (0–30 metres depth) salinity in the Sicily Channel and the mixed layer depth in the Aegean Sea, Adriatic Sea, and Gulf of Lions (the regression explains 72%, 32% and 86% of data variance, respectively, N = 336) (Fig. 3). This is in good agreement with previous studies based on laboratory experiments^24^ and energy budget analysis^25^,^26^. These studies have shown that in a semi-enclosed basin, an increase in the surface buoyancy loss, such as the one required to increase the mixed layer depth in the Mediterranean sub-basins, implies an increase in the energy inflow at the basin entrance. In that situation, a more energetic inflow implies that the Atlantic water would reach the Strait of Sicily earlier, due to the enhancement of the thermohaline circulation. The shorter Atlantic water transition time to the Sicily Channel would mean less time to mix with the surrounding resident water and thus the arrival of fresher surface water.

## Discussion

### Mediterranean circulation perturbations

**Figure 4:**
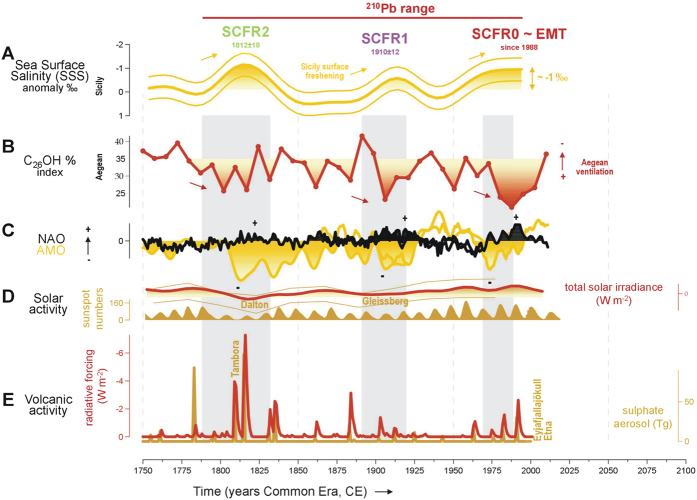
Eastern Mediterranean Transient (EMT) and SCFR events over the last two centuries (within the ^210^Pb range interval). Time progression in years of the Common Era (CE). (**A**) The 20-year Gaussian low pass filter of the normalized δ^18^O_sw_ composite is shown (values above average are filled in) in comparison with (**B**) the n-hexacosan-1-ol index, as a proxy for deep ventilation in the Aegean (this study) (higher ventilation downwards). (**C**) NAO and AMO indices, both instrumental and reconstructed^30^,^31^. (**D**) Total solar irradiance with 1-sigma uncertainty in watts per square meter (right axis; shown as the difference of total solar irradiance from the value of the Physikalisch-Metorologisches Observatorium Davos composite during the solar cycle minimum of the year 1986, i.e. 1365.57 watts per square)^40^ and annual total sunspot numbers (left axis) as provided by the Royal Observatory of Belgium, Sunspot Index Data center (http://www.sidc.be/silso/datafiles); note the three periods of low solar activity: Dalton minimum from 1790 to 1830, Gleissberg minimum around the 1900s and a subdued minimum over the 1970s. (**E**) Two estimations for explosive volcanism: global total injection of sulphate aerosol (teragrams; right axis)^41^ and annual-mean volcanic radiative forcing (watts per square meter; left axis) derived from aerosol optical depth data (a measure of stratospheric transparency to incoming solar radiation; ref. 63), which has been used for the volcanic-only forcing LOVECLIM simulation performed for the global compilation of marine sediment reconstructions over the past two millennia^64^. The Eastern Mediterranean Transient (EMT, since 1988) event and SCFR anomalies are shown by grey-shaded bands (SCFR1 1910 ± 12 and SCFR2 1812 ± 18). Note that the lightest peaks in δ^18^O_sw_ are in line with a decrease in solar irradiance, increase in volcanic activity, positive NAO phases and are preceded by negative AMO phases and higher deep ventilation in the Aegean.

This study reveals a recurrent δ^18^O_sw_ pattern of SCFR events in the upper layer of the Strait of Sicily (Fig. 4A). The model used to test these results suggests a link between SCFR events and enhanced deep-water formation (Fig. 3). The organic chemical index reflecting oxygenation variability identifies an exceptional episode of deep-water ventilation in the Aegean Sea during the SCFR0 ~ EMT, since 1988 (Fig. 4B), detected within the industrial period with time uncertainties below 20 years (SI Appendix, Table S1). Note the occurrence of SCFR1 and SCFR2, in 1910 ± 12 and 1812 ± 18 respectively, which are of weaker intensity than the EMT (Fig. 4A,B). Increased deep-water formation may feed the overturning circulation intensification and this in turn can cause a faster flow of Atlantic surface water that would arrive to Sicily less mixed and thus fresher. Sensitivity experiments from a numerical model emphasized the linear correlation between the amount of deep-water formation and the zonal overturning circulation strength in the Mediterranean Sea^27^, while no evidence of the wind stress influence on the temporal variability of the water fluxes in the Sicily Strait has been found^28^. The multi-decadal Sicily Channel freshening pattern may have also been affected by variations of the salt content in the inflowing Atlantic water at the Gibraltar Strait, but no significant changes can be seen in Alboran Sea surface water properties before, during and after the EMT^29^.

Recently, the influence of the Adriatic-Ionian Bimodal Oscillating System (BiOS) has been shown on decadal-scale salinity variations in Sicily Channel intermediate waters^9^. Depending on the phase of the BiOS, surface water crossing the Sicily Channel is directed towards the North Ionian or the Levantine Basin. This affects the salt content in the Levantine Basin and the production of dense water in the Aegean^10^. However, there is no direct effect on the salinity of the upper layer in the Sicily Channel. In fact, the signature of the BiOS in the Sicily Channel is expected at intermediate waters with salinity changes lower than 0.1 psu at a quasi-decadal periodicity^9^.

The analysis of temperature and salinity inversions in the Eastern Mediterranean historical observations suggests that no EMT-like events occurred back to 1910^15^. This is compatible with our results, which show a destabilisation of the Mediterranean overturning circulation in 1910 ± 12 (SCFR1, Fig. 4A,B). Roether *et al.*^15^ assumed that past Mediterranean circulation anomalies led to the same temperature/salinity signatures, with similar surrounding hydrographic processes (i.e. Aegean deep-water outflow) and duration (about 20 years) than the most recent EMT. Our study widens this perspective, takes into account different deep-water formation sites and highlights the possibility that perturbation episodes happened prior to the 1910 pioneer Danish oceanographic expedition.

### Past EMT-type events? Climate forcings and feedbacks

The EMT impact on Sicily Channel hydrography was recently identified through the arrival of Mediterranean intermediate waters with high salinity and potential density in contrast to the overlying surface waters, where a pronounced salinity minimum was maintained^20^. The surface-to-deep contrast in the Sicily Channel sedimentary record is identified for the EMT as well as for previous freshening episodes (Fig. 4A,B). The marked stratification in the Sicily Channel water column (Fig. 4A) associated with enhanced Mediterranean deep-water formation is strongly suggestive of recurrent modifications in the thermohaline circulation pattern in historical time, with multi-decadal-scale perturbations from steady state. We cannot disentangle the single contribution of deep-water formation sources and specifically the contribution of the deep-water formation in the Aegean Sea, which was arguably the most peculiar EMT feature^2^,^3^,^4^. However, episodes of enhanced deep-water ventilation in the Aegean Sea are fully consistent with SCFR0 ~ EMT, SCFR1 and SCFR2 (Fig. 4B), supporting the hypothesis of the significant role of the Eastern Mediterranean in circulation changes from a steady state.

A variety of oceanographic processes occurred within the Eastern basin and favoured the ‘transient’ onset^2^,^3^,^4^. Specifically, the increasing salinity of intermediate water in the Cretan Sea seems to be a recurrent process favoured by the cyclonic circulation in the North Ionian Gyre^9^,^10^. The accumulation of saline and dense water has occurred three times over the past fifty years^10^ that culminated in a pronounced deep-water formation in the Aegean Sea only during the EMT, due to the exceptional 1987, 1992 and 1993 winter heat fluxes in the region^6^,^22^. Thus it is very likely that Cretan Sea processes together with peculiar Aegean Sea atmospheric circulation led to previous Mediterranean overturning circulation perturbations and/or EMT-like episodes. The comparison between the composite of the Sicily Channel δ^18^O_sw_ profiles (Fig. 4A), northern Atlantic atmospheric circulation patterns and oceanic climate indices (refs 30,31; Fig. 4C) points to the coupling between freshening episodes, positive North Atlantic Oscillation (NAO) and negative Atlantic Multidecadal Oscillation (AMO) phases. The NAO is the most prominent mode of variability in the Northern Hemisphere winter climate and, while its dynamics at inter-annual scale can be explained as a purely internal pattern of atmospheric circulation, its inter-decadal variability may be influenced by oceanic and sea-ice changes^32^. The AMO index is useful in this regard given that it captures the fluctuation in detrended Atlantic SST anomalies north of the equator and its ‘engine’ involves the North Atlantic thermohaline circulation, which ultimately is connected with Mediterranean convection processes^33^.

The effects of large-scale atmospheric circulation over the Aegean Sea region could have played a role in the EMT origin^34^,^35^. The NAO index increased from −0.1 to 0.6 during the EMT (Fig. 4C). The positive NAO phase may have increased the net Aegean evaporation, reduced the river runoff into the Black Sea^34^, and simultaneously strengthened the cyclonic activity over the Eastern Mediterranean, thereby enhancing the southward advection of anomalous cold air over the Aegean^36^. Recently Josey *et al.*^37^ suggested that the NAO Index alone is not a sufficient predictor to account for significant surface heat flux variability in the Mediterranean deep-water formation sites. This is especially true in the Aegean Sea where only the positive state of the East Atlantic/West Russian (EA/WR) pattern results in strongly enhanced heat loss over this region^37^. The co-variability between the NAO and EA/WR indices was found to exhibit large decadal to century variations, indicating that climate variability over the continent is temporarily decoupled from the NAO^38^. For autumn and winter, while deep Mediterranean water is formed, the correlations between the NAO and the EA/WR indices were mostly positive, suggesting that strong (weak) westerlies over the North Atlantic tend to coincide with strong (weak) anomalous northerly flow over central Europe and the Mediterranean^38^. In addition to the possible direct impact on winter Aegean Sea surface heat fluxes, both NAO and AMO are of importance for the Mediterranean climate variability^39^. In particular, at multidedacal scales, positive NAO phases lead to decreased precipitation (i.e. higher salinity) while negative AMO leads to reduced SSTs. Both processes may have favoured deep-water formation events on relatively long time scales.

Thermohaline circulation destabilisations in the Mediterranean circulation also seem to be linked to reduced solar activity (ref. 40; Fig. 4D) and to frequent volcanic eruptions (ref. 41; Fig. 4E). Solar activity modulates patterns in surface temperature and pressure that resemble NAO phases, through dynamical coupling processes between the stratosphere and the troposphere that transmit the solar signal to the Earth’s surface^42^,^43^. The increase in sulphur aerosols from tropical volcanic emissions produces stratospheric and surface conditions that resemble the positive NAO phase^44^ and cause decrease in oceanic heat content, with long-lived temperature anomalies extending to the mid-depth and deep ocean, an increase in sea ice volume and enhancement in the overturning circulation of the North Atlantic Ocean following these eruptions^45^. These phenomena may have shaped the North Atlantic atmospheric pattern, which in turn may have led to Aegean SSTs cooling and thus surface buoyancy loss and enhanced Eastern Mediterranean deep-water formation during EMT-like events.

## Materials and Methods

The ^210^Pb_excess_ profile presents an exponential decay of unsupported ^210^Pb down to ~10 cm depth in St 342 and Athos-M2, 8 cm in St 407, and from then on, total ^210^Pb activities fall to virtually constant background values (SI Appendix, Fig. S1), indicating low levels of bioturbation and a shallow mixed layer. Sediment accumulation rates were determined by the slope of the least squares fit for the natural log of the ^210^Pb_excess_ activity versus depth. The ^210^Pb results suggest a sedimentation rate of ~55–90 cm per millennium for the uppermost sediment cores. Two different models were used, depending on the regional depositional setting^46^. The model used is constant flux – constant sedimentation for Sicily Channel cores and constant rate of supply (CRS) for the Aegean Sea Athos-M2 core. ^210^Pb activities were measured via alpha counting of its daughter, ^210^Po, assuming secular equilibrium between the two isotopes. In St 342 and St 407 cores, ^210^Po was extracted from the sediment with hot HNO_3_ and H_2_O_2_, and spiked with ^209^Po (NIST standard SRM 4326, diluted to 0.43 Bq g^−1^) used as a yield monitor. After separation of the leachate from the residue, the solution was evaporated to near dryness and the nitric acid was eliminated using concentrated HCl. The residue was dissolved in 1.5 N HCl. Iron was reduced using ascorbic acid and Po was plated onto a silver disk overnight, at room temperature^47^. Concerning the ^226^Ra activity, the lowest values of total ^210^Pb in the cores were considered. A successful intercalibration of ^210^Pb analyses has been carried out and the analyses of the same sample with the two different ^209^Po internal standards used at CNR-ISMAR (Bologna) and MSRC (Stony Brook) have yielded nearly identical results. Precision calculated from three independent analyses is ±4.6%. In the Athos-M2 core, dried sediments were leached successively with HNO_3_, HNO_3_-HClO_4_, HF and HCl and ^210^Po isotopes were deposited on silver discs and counted on a total a-counter (Ortec EG&G)^48^. Repeated measurements of a number of sediment samples showed analytical precision better than 5%. The ^226^Ra-supported ^210^Pb (background) was assumed to be 36Bq/kg, a value measured in the deeper part of the core (36 cm). Five accelerator mass spectrometry ^14^C dates were performed at the laboratories of Beta Analytic (USA) on cleaned hand-picked planktonic foraminifera from the Athos-M2 core. ^14^C ages were converted into calibrated ages using the Calib vs. 7.02 software^49^ and the MARINE13 calibration dataset, with local ΔR = 58 ± 85 years correction^50^. Sedimentation rates derived from the uppermost calibrated ages and from the CRS are very close one each other, respectively 87 and 83 cm/kyr^16^.

Stable isotopes were measured on carbonate shells of *Globigerinoides ruber* and *Uvigerina peregrina* by an automated continuous flow carbonate preparation GasBenchII device and a ThermoElectron Delta Plus XP mass spectrometer at the IAMC-CNR (Naples) isotope geochemistry laboratory. Acidification of samples was performed at 50 °C. Every 6 samples, an internal standard (Carrara Marble with δ^18^O = −2.43‰ versus VPDB and δ^13^C = 2.43‰ vs. VPDB) was run and every 5 samples the NBS19 international standard was measured. Standard deviations (1σ) of carbon and oxygen isotope measures were estimated at 0.08 and 0.11‰, respectively, on the basis of ~5 samples measured three times. All the isotope data are reported in δ‰ versus VPDB.

Foraminiferal shells were picked from the size fraction 250–350 μm and then gently crushed between methanol-cleaned glass microscope slides. Afterwards foraminiferal samples were cleaned using a standard method^51^ that has been slightly modified to include a reductive step. Cleaned samples were dissolved in 1% HNO_3_. An aliquot of the samples was injected into an Inductively Coupled Plasma – Mass Spectrometer (ICP-MS, Agilent model 7500ce) for preliminary calcium (Ca^2+^) determination. The remaining solutions were diluted to 40 ppm [Ca^2+^] and then injected into the ICP-MS to obtain [Ca^2+^], magnesium [Mg^2+^], and other trace metal concentrations. The long-term reproducibility of the Mg/Ca analyses yields an analytical precision of 1.3%, which was obtained by performing repeated measurements of two in-house consistency standards with variable metal/Ca ratios to mimic the natural variations in foraminifera (N = 158). A commonly used limestone standard (ECRM752–1) with a specified Mg/Ca of 3.75 mmol mol^−1^ was analyzed to evaluate inter-laboratory consistency, yielding values of 3.70 ± 0.09 mmol.mol^−1^. Al/Ca and Mn/Ca were analyzed to monitor the presence of any remaining clays or Mn-coatings after the cleaning procedure^51^,^52^. Mn/Ca values in samples from both sites were all well below the commonly used thresholds, as well as the Al/Ca concentrations at St 342^51^,^52^. However, Al/Ca values from St 407 were largely above the threshold, and were well correlated with Mg/Ca measurements (r^2^ = 0.6). Therefore, Mg/Ca values were corrected^52^, and in this study we only refer to the corrected Mg/Ca values for St 407. Finally, equation (53) (Mg/Ca = 0.449exp(0.09T)) was selected for converting the *Globigerinoides ruber* Mg/Ca values for both sites into calcification temperatures, due to closest correspondence with modern temperature values for the depth and season of this species.

Uncertainties associated with the δ^18^O_sw_ record for cores St 407 and St 342 are estimated by propagating the uncertainties associated with the analytical protocols (see above), with conversion of *G. ruber* Mg/Ca values to calcification temperatures^53^, and with the δ^18^O_calcite_ paleotemperature equation used to account for the temperature-dependent water-to-carbonate isotopic fractionation^54^. We conducted an error propagation exercise performed for core St 342, for which the Mg/Ca correction due to high Al/Ca values was not necessary (see above). The propagated uncertainty estimated for each δ^18^O_sw_ data point at this site amounts to ±0.24‰ and ±0.5‰ at 68% (1σ) and 95% (2σ) confidence levels, respectively. If errors propagate through a δ^18^O_sw_ shift between two individual data points the 1σ (2σ) uncertainty of δ^18^O_sw_ then amounts to ±0.3‰ (±0.7‰). The uncertainty associated with the δ^18^O_sw_ composite was quantified by dividing the uncertainty estimated for the δ^18^O_sw_ records by their standard deviation.

Throughout the sediment cores studied, two groups of fossil organic compounds have been identified and quantified: alken-*2*-ones, which are constituents of the autochthonous biomass synthesized by the coccolithophore flora^55^ and *n*-hexacosan-*1*-ol and *n*-nonacosane, as allochthonous vascular plant debris^56^. Sediment samples (~2 grams) were freeze-dried (12 hours; Edwards High Vacuum International) and extracted by sonication and vortex shaking, using 7.5 mL of dichloromethane (15 minutes; Selecta). The process was repeated three times to ensure that the extraction yield would be higher than 90%. The extracts were dried under nitrogen and hydrolysed with 6% potassium hydroxide in methanol to avoid interferences in detection and quantification of the compounds (2 ml; 12 hours; room temperature). Extraction with hexane (2 ml; three times) yielded a fraction enriched in neutral compounds, which was cleaned with ultra-pure water. Extracts were derived with bis(trimethylsilyl)trifluoroacetamide (12 hours; room temperature). Samples were injected with toluene. They were analysed with a Varian gas chromatograph Model 3800 equipped with a septum programmable injector and a flame ionisation detector. The instrument was equipped with a CPSIL-5 CB column coated with 100% dimethylsiloxane (film thickness of 0.12 μm). Hydrogen was the carrier gas (2.5 ml/min). Oven temperature was programmed from 90° (holding time of 1 min) to 170 °C at 20 °C/min, then to 280 °C at 6 °C/min (holding time 25 min), and finally, to 315 °C at 10 °C/min (holding time of 12 min). The injector was programmed from 90 °C (holding time of 0.5 min) to 310 °C at 200 °C/min (final holding time was 55 min). Absolute concentration errors were below 10% (resolution between the alkenones from 1.5 to 1.7). Reproducibility tests showed that uncertainty in the alkenone unsaturation index determinations was lower than 0.0165 (ca. ±0.5 °C). Temperatures were derived from a global core top calibration providing alkenone production temperatures^57^.

The NEMOMED8 regional hindcast is obtained from a simulation of the NEMO model^58^. The model has a spatial resolution of 1/8° in the horizontal and 43 z-levels inhomogeneously distributed (i.e higher resolution near the surface). The model is initialized using the MEDATLAS climatology and a 15-year spin up. After the spin-up, the model is run for the period 1961–2000 forced by daily atmospheric fluxes from ARPERA. ARPERA is a dynamical downscaling of the ERA40 reanalysis (resolution of 125 km) by the regional climate model ARPEGE-Climate (grid stretched over the Mediterranean Sea, resolution of 50 km). A restoring term to observed SST is added to the net surface heat flux from ARPERA, in order to improve the air-sea interaction. The mixed layer depth has been estimated as the depth where the difference with surface density is larger than 0.01 kg/m^3^. Model monthly outputs have been averaged to form seasonal and annual quantities.

### Data availability

Supplementary data are available at https://doi.org/10.1594/PANGAEA.861972, https://doi.org/10.1594/PANGAEA.861973 and at https://doi.org/10.1594/PANGAEA.861974.

## Additional Information

**How to cite this article**: Incarbona, A. *et al.* Mediterranean circulation perturbations over the last five centuries: Relevance to past Eastern Mediterranean Transient-type events. *Sci. Rep.* **6**, 29623; doi: 10.1038/srep29623 (2016).

## Supplementary Material

Supplementary Information

## Figures and Tables

**Figure 1:**
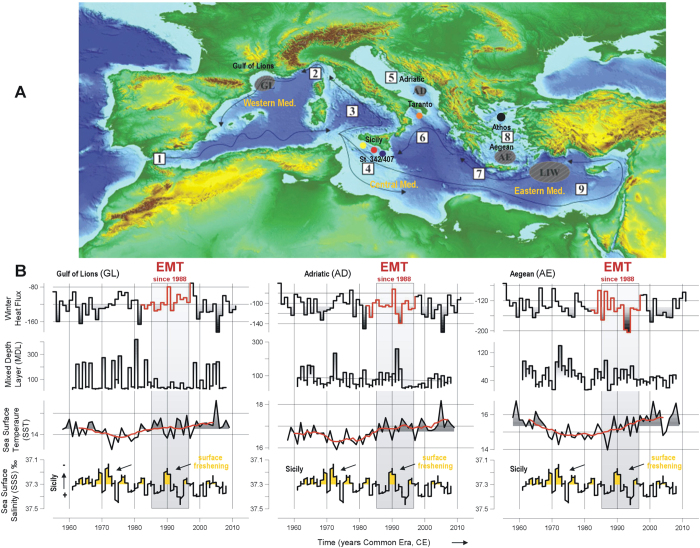
(**A**) Bathymetric map of the Mediterranean Sea (from Ocean Data View, version ODV 4.7.4, https://odv.awi.de/), showing location of the sites analysed for this study: Sicily Channel box-cores St 342 (36°42’N, 13°55’E, 858 m depth; red dot) and St 407 (36°23’N, 14°27’E, 345 m depth; blue dot) and the Aegean Sea multicore Athos-M2 (40°5’N, 24°33’E, 1018 m depth; black dot). Black arrows show the path of surface water circulation and white squares with numbers refer to 1-Gibraltar Strait, 2-Ligurian Basin, 3-Tyrrhenian Basin, 4-Sicily Channel, 5-Adriatic Basin, 6-Adriatic-Ionian Bimodal Oscillating System^9^, 7-Cretan Channel, 8-Aegean Basin and 9-Levantine Basin. Dashed dark grey areas show sites of deep and intermediate water formation: GL, Gulf of Lions; AD, southern Adriatic Sea; AE, Aegean Sea; LIW, Levantine Intermediate Water. Other sites previously studied are the Ocean Drilling Program Site 963 (yellow dot; ref. 59) and cores in Taranto (GT89-3, GT91-3, NU04 and GeoB 10709-4; orange dot; refs 60,61). (**B**) Evolution of key diagnostics from the NEMOMED8 hindcast since the 1960s during winter months, when deep water formation takes place: winter heat flux and mixed depth layer (MDL) in the Aegean, the South Adriatic and the Gulf of Lions regions. The time evolution of sea surface temperatures (SST; MEDAR/MEDATLAS2002 database; ref. 62) in these areas and sea surface salinity in the Sicily Channel are also shown. The Eastern Mediterranean Transient (EMT) is shaded grey.
