# Supplementary material for: Mediterranean circulation perturbations over the last five centuries: Relevance to past Eastern Mediterranean Transient-type events
Source: Sci Rep. 2016 Jul 14;6:29623. doi: 10.1038/srep29623 (PMC4944156; doi:10.1038/srep29623)
Supplement: Supplementary Information [file srep29623-s1.pdf]

Supplementary information for

Mediterranean circulation perturbations over the last five centuries: Relevance to past Eastern Mediterranean Transient-type events

Authors

Alessandro Incarbona[a,1†], Belen Martrat[b,c†], P. Graham Mortyn[d,e†], Mario Sprovieri[f†], Patrizia Ziveri[d,g,h†], Alexandra Gogou[i], Gabriel Jordà[j], Elena Xoplaki[k], Juerg Luterbacher[k,l], Leonardo Langone[m], Gianluca Marino[d,n], Laura Rodríguez-Sanz[d,n], Maria Triantaphyllou[o], Enrico Di Stefano[a], Joan O. Grimalt[b], Giorgio Tranchida[f], Rodolfo Sprovieri[a], Salvatore Mazzola[f]

Author affiliation

[a] Università di Palermo, Dipartimento di Scienze della Terra e del Mare, Via Archirafi 22, 90123 Palermo, Italy; [b] Department of Environmental Chemistry, Institute of Environmental Assessment and Water Research (IDÆA), Spanish Council for Scientific Research (CSIC), Jordi Girona 18, 08034 Barcelona, Spain; [c] University of Cambridge, Department of Earth Sciences, Downing Site, Downing Street, Cambridge CB2 3EQ, United Kingdom; [d] Universitat Autònoma de Barcelona (UAB), Institute of Environmental Science and Technology (ICTA), Edifici Z, Carrer de les Columnes, Campus de la UAB, 08193 Bellaterra (Cerdanyola del Vallès), Barcelona, Spain; [e] UAB, Department of Geography, 08193 Bellaterra (Cerdanyola del Vallès), Barcelona, Spain; [f] Consiglio Nazionale delle Ricerche (CNR), Istituto per l'Ambiente Marino Costiero, Via del Mare 3, 91021 Torretta-Granitola (Trapani), Italy; [g] Vrije Universiteit Amsterdam, Department of Earth Sciences, Faculty of Earth and Life Sciences, de Boelelaan 1085, 1081HV Amsterdam, The Netherlands; [h] ICREA, Catalan Institution for Research and Advanced Studies, 08010, Barcelona, Spain; [i] Hellenic Centre for Marine Research (HCMR), Institute of Oceanography, P.O. Box 712, 19013 Anavyssos, Greece; [j] Department of Ecology and Marine Resources, IMEDEA (CSIC-UIB), Institut Mediterrani d'Estudis Avançats, Miquel Marquès 21, 07190 Esporles, Illes Balears, Spain; [k] Justus-Liebig-University Giessen, Department of Geography, Climatology, Climate Dynamics and Climate Change, Senckenbergstr. 1, 35390 Giessen, Germany; [l] Centre for International Development and Environmental Research, Justus-Liebig-University Giessen, 35390 Giessen, Germany; [m] CNR, Istituto di Scienze Marine, Via Gobetti 101, 40129 Bologna, Italy; [n] Research School of Earth Sciences, The Australian National University, Canberra, Australian Capital Territory 2601, Australia; [o] University of Athens, Faculty of Geology and Geoenvironment, Department of Historical Geology – Paleontology, Panepistimiopolis 15784, Athens, Greece.

[†] These authors contributed equally to this work.

[1] To whom correspondence should be addressed: A. I. (alessandro.incarbona@unipa.it).

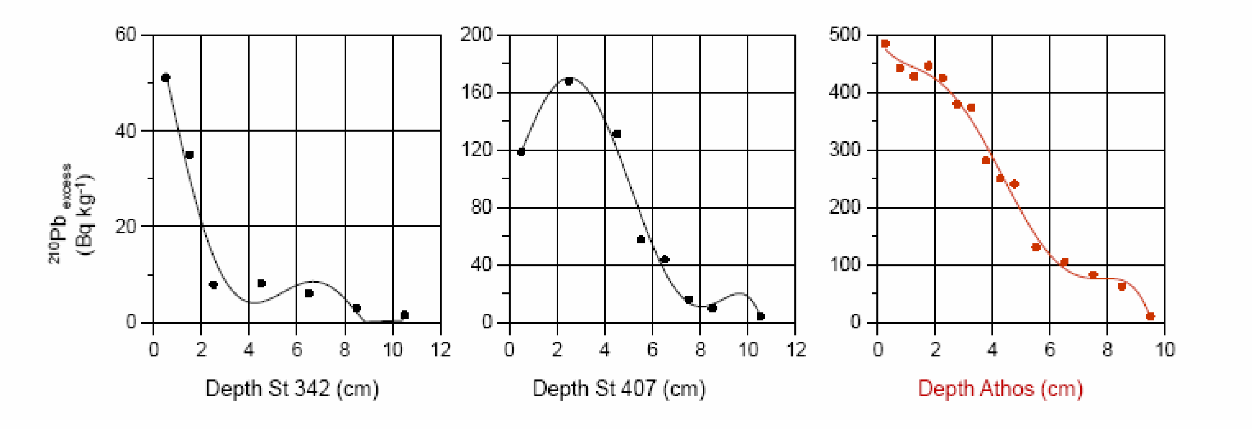

Figure S1

Age control for St 342, St 407 (Sicily) and Athos-M2 (Aegean) during the industrial period. $^{210}Pb_{excess}$ versus depth.

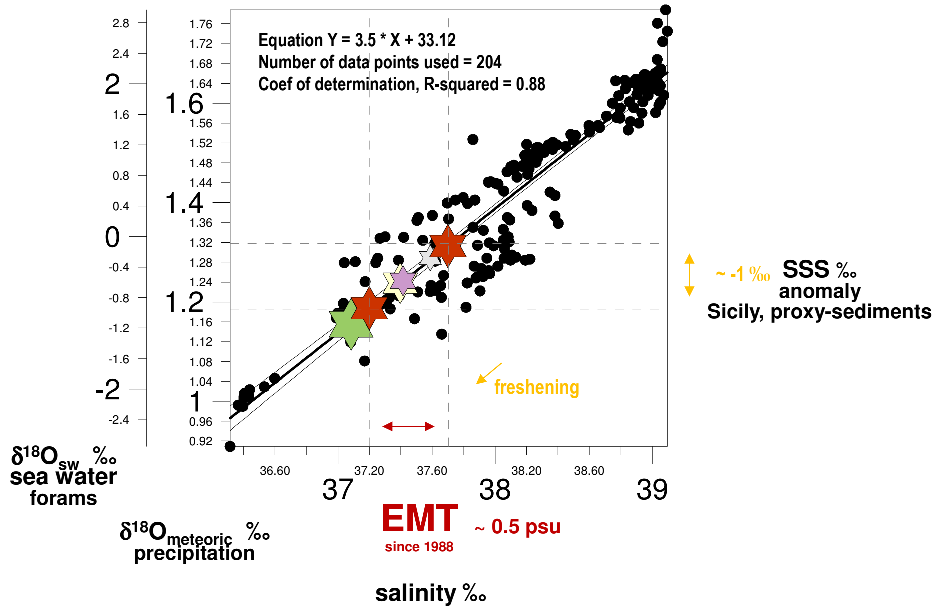

Figure S2

The $\delta^{18}O_{sw}$ record documents a multi-decadal period of Sicily Channel surface freshening episodes.

In the Mediterranean basin, the correlation coefficient between $\delta^{18}O_{precipitation}$ in meteoric precipitation (IAEA/WMO. Global Network of Isotopes in Precipitation. The GNIP Database. Accessible at: http://www.iaea.org/water, 2013) and salinity (MEDAR Group. MEDATLAS/2002 database. IFREMER Edition, 4 CDs, 2002) is up to 0.88. The EMT since 1988 is the reference climatic event in the circulation and water mass properties in the last century. In the Sicily Channel, it featured a 0.5 psu salinity change (red symbols; from 37.7 to 37.2 ‰; 20), revealed in our records by a ~ -1‰ $\delta^{18}O_{sw}$ anomaly, approximately equivalent to a 0.1‰ decrease in the $\delta^{18}O_{precipitation}$ (from 1.3 to 1.2‰). The reconstructed water $\delta^{18}O_{sw}$ *G. ruber* (corrected by using Mg/Ca and alkenone temperature profiles) averages ~ -0.1‰ over the last five centuries. Taking this value as a reference, freshening anomalies are provided as more- or less-pronounced than the most recent EMT: slightly less than 0.5 salinity change for SCFR1 ca.1910 (in purple), SCFR3 ca. 1725 (in pale yellow), SCFR4 ca. 1580 (in grey), and higher than 0.5 for SCFR2 ca. 1812 (in green).

	depth	Pb-210 total	Pb-210 xs
	(cm below sea floor)	(Bq kg^{-1})	(Bq kg^{-1})
Sicily, St 342 0-1	0.500	66	51
Sicily, St 342 1-2	1.500	50	35
Sicily, St 342 2-3	2.500	23	8
Sicily, St 342 4-5	4.500	23	8
Sicily, St 342 6-7	6.500	21	6
Sicily, St 342 8-9	8.500	18	3
Sicily, St 342 10-11	10.500	17	2
Sicily, St 342 12-13	12.500	15	-
Sicily, St 342 14-15	14.500	14	-
Sicily, St 342 16-17	16.500	15	-
Sicily, St 342 24-25	24.500	16	-
Sicily, St 407 0-1	0.500	133	119
Sicily, St 407 2-3	2.500	182	168
Sicily, St 407 4-5	4.500	145	131
Sicily, St 407 5-6	5.500	72	58
Sicily, St 407 6-7	6.500	58	44
Sicily, St 407 7-8	7.500	30	16
Sicily, St 407 8-9	8.500	24	10
Sicily, St 407 10-11	10.500	19	5
Sicily, St 407 12-13	12.500	14	-
Sicily, St 407 14-15	14.500	14	-
Sicily, St 407 16-17	16.500	13	-
Sicily, St 407 24-25	24.500	14	-
Aegean, Athos (M2)	0.250	523	485
Aegean, Athos (M2)	0.750	480	442
Aegean, Athos (M2)	1.250	466	428
Aegean, Athos (M2)	1.750	484	446
Aegean, Athos (M2)	2.250	463	425
Aegean, Athos (M2)	2.750	417	379
Aegean, Athos (M2)	3.250	412	374
Aegean, Athos (M2)	3.750	319	281
Aegean, Athos (M2)	4.250	288	250
Aegean, Athos (M2)	4.750	279	241
Aegean, Athos (M2)	5.500	169	131
Aegean, Athos (M2)	6.500	143	105
Aegean, Athos (M2)	7.500	121	83
Aegean, Athos (M2)	8.500	101	63
Aegean, Athos (M2)	9.500	48	10
Aegean, Athos (M2)	10.500	38	-

site	depth cm	material	^{14}C age	cal year BP	error ± 1σ	age BCE/CE
Athos	16.5-18.5	foram.	200±30	200	21	1750
Athos	23.5-25.5	foram.	760±30	355	60	1595

Table S1

Age controls for St 342, St 407 and Athos-M2. ^{210}Pb measurements for St 342 and St 407 were performed at the Consiglio Nazionale delle Ricerche (Bologna, Italy) and for Athos-M2 at the Hellenic Centre for Marine Research (Athens, Greece). The accelerator mass spectrometry radiocarbon (^{14}C) dates were performed at the laboratories of Beta Analytic (Miami, USA) on cleaned, hand-picked planktonic foraminifera and converted to calendar age using a reservoir age of 400 years and the CALIB version 7.02 software (49) and the MARINE 13 calibration dataset, with local $\Delta R = 58 \pm 85$ years (50).
